# Interplay among Vaginal Microbiome, Immune Response and Sexually Transmitted Viral Infections

**DOI:** 10.3390/ijms20020266

**Published:** 2019-01-11

**Authors:** Maria Gabriella Torcia

**Affiliations:** Department of Clinical and Experimental Medicine, University of Firenze, 50139 Firenze, Italy; maria.torcia@unifi.it; Tel.: +39-0552758227

**Keywords:** vaginal microbiota, HIV, HPV, HSV2, cytokines, chemokines, innate immunity, adaptive immunity

## Abstract

The vaginal ecosystem is important for women’s health and for a successful reproductive life, and an optimal host-microbial interaction is required for the maintenance of eubiosis. The vaginal microbiota is dominated by *Lactobacillus* species in the majority of women. Loss of *Lactobacillus* dominance promotes the colonization by anaerobic bacterial species with an increase in microbial diversity. Vaginal dysbiosis is a very frequent condition which affects the immune homeostasis, inducing a rupture in the epithelial barrier and favoring infection by sexually transmitted pathogens. In this review, we describe the known interactions among immune cells and microbial commensals which govern health or disease status. Particular attention is given to microbiota compositions which, through interplay with immune cells, facilitate the establishment of viral infections, such as Human Immunodeficiency Virus (HIV), Human Papilloma Virus (HPV), Herpes Simplex Virus 2 (HSV2).

## 1. The Vaginal Ecosystem

The vaginal mucosal ecosystem is comprised of a stratified squamous epithelium covered by a mucosal layer continuously lubricated by cervicovaginal fluid (CVF), which contains products of epithelial cells, such as mucins and antimicrobial molecules B-Defensin, Lipocalin, Elafine and secretory leukocyte protease inhibitor (SLPI) [1,2], IgA and IgG antibodies produced by mucosal plasma cells. CVF continuously lubricates epithelium, maintains the fluidity of the ecosystem and represents the first line of defense against exogenous pathogen colonization through the activity of mucins which entrap microbes and facilitate their binding to antibodies [3].

Cervicovaginal fluid also helps to contain the vaginal microbiota, microbial communities which exist in a mutualistic relationship with the host. The vaginal microbiota is unique in that, in many women, it is most often dominated by *Lactobacillus* species [4,5]. The latter produce lactic acid and bacteriocins, which contribute to prevent bacterial growth and, by maintaining a low vaginal pH (3.0–4.5), eventually favor *Lactobacillus* dominance. Moreover, *Lactobacillus* species adhere to epithelial surfaces, preventing the adhesion of bacteria able to infect epithelial cells, promote the autophagy of cells infected by viruses, bacteria or protozoa, facilitating their elimination [6], and modulate any inflammatory process that could have negative consequences, especially during pregnancy [7].

These activities are also necessary to prevent immune reactions against sperm. It is believed that the human behavior with sexual activities at any time during the menstrual cycle, as well as during gestation, may have contributed to select *Lactobacillus* species in the vaginal microbiota.

The detailed composition of vaginal microbiota and the relative abundance of the bacterial species has recently been defined through high-throughput 16s rRNA gene sequencing. At least five microbial communities, here referred as Community State Type (CST) were identified. Four CSTs are dominated by *Lactobacillus* (*L.*) species highly adapted to the vaginal environment [4,5]. In particular CST-I is dominated by *Lactobacillus crispatus*; CST-II by *Lactobacillus gasseri*; CST-III by *Lactobacillus iners*; CST-V by *Lactobacillus jensenii*. The CST-IV is comprised by polymicrobial communities, with prevalence of strictly anaerobic species of the order *Gardnerella*, *Atopobium*, *Mobiluncus*, *Megasphoera Prevotella*, *Streptococcus*, *Mycoplasma*, *Ureaplasma*, *Dialister*, *Bacteroides*, etc. [4,8,9]. The latter CST is very common in black and Hispanic women, ranging from 10% in United States [4] to 60% in South Africa [10]. The factors driving racial and geographic differences in cervicovaginal communities are not known yet, but could include changes in sexual behavior, hygienic practices, rectal colonization or even host genetics [11].

In many cases, a low vaginal pH may be maintained by microbes of CST-IV through the production of lactic acid [9], in this case, the presence of CST-IV is completely asymptomatic. However, loss of *Lactobacillus* dominance facilitates Bacterial Vaginosis (BV), the most common vaginal infection in reproductive-aged women [12,13], characterized by vaginal discharge, irritation, fishy odor and a vaginal pH often >4.5. Hormonal changes strongly affect the composition of vaginal microbiota during a woman’s life, in particular at menopausal age, when reduced estrogen levels decrease the glycogen availability with the consequent depletion in *Lactobacillus* species [14].

In addition to epithelial cells and microbiota, the vaginal ecosystem comprises also cells of innate and adaptive immunity as neutrophils, macrophages, classic dendritic cells, Langerhans cells, NK cells, T and B lymphocytes. [15]. CD3^+^ T lymphocytes, predominantly CD8^+^ lymphocytes, are distributed in the lamina propria of the cervix and vagina. In particular, in ectocervical mucosa they have an effector/memory (CD27^−^ CD45RA^−^) or effector phenotype (CD27^−^CD45RA^+^); B cells are found as aggregates or follicular-like structures surrounded by T lymphocytes. Plasma cells, as well as a small number of macrophages CD68^+^ and dendritic cells, are distributed throughout the lamina propria. Dendritic cells and monocyte/macrophagic CD14^+^ cells represent the most prevalent antigen-presenting cells in the vaginal ecosystem. Immune cell trafficking and activation throughout the reproductive tract is tightly mediated by the expression of pattern recognition receptors (PRR) [16,17] and is regulated by endocrine signaling [18].

## 2. Interplay between Host and Vaginal Microbiota in Health and Disease

Interplay between the cervicovaginal microbiota and the cells of immune system is determinant to prevent infections by external pathogens and to maintain an immuno-tolerant environment, particularly during pregnancy. Sex hormones are key regulators of this interplay: they regulate the production of antimicrobial peptides (beta-defensin, alpha Defensin, SLPI) and of pro-inflammatory cytokines (IL-6, IL-8) by the vaginal epithelial cells to ensure, when necessary, survival of sperm and to prevent ascending infections [19]; estrogens in particular also contribute to select microbial populations. At prepuberal age, vaginal microbiota is dominated by anaerobic species of the *Enterobacteriacee* and/or *Staphylococcacee* family. At puberty, the increased concentrations of estrogens promote the accumulation of glycogen by mature epithelial cells. Maltotriose and alpha-dextrins, derived from alpha-amylase-mediated glycogen digestion are selective food for *Lactobacillus* species that use these products for the synthesis of lactic acid. The low pH, the maintenance of mucous viscosity and the prevention of bacterial binding on epithelial surfaces are all factors that favor *Lactobacillus* dominance [20].

When *Lactobacillus* dominance is lost and microbial diversity increases, changes in immune and epithelial homeostasis often appear, induced through multiple mechanisms, as: (a) production of pro-inflammatory cytokines and chemokines, (b) recruitment of immune cells (c) reduction in the viscosity of the CVF, due to the production of mucin-degrading enzymes (including sialidase, α-fucosidase, α-and β-galactosidase, *N*-acetyl-glucosaminidase, and glycine and arginine aminopeptidases [21,22]. Physical/chemical changes in vaginal ecosystem ultimately affect the barrier properties of both CVF and the genital epithelium and increase the risk of infection with sexually transmitted pathogens. In fact, vaginal dysbiosis was associated with increased risk of acquisition of Sexual Transmitted Infections (STI) such as *Neisseria gonorrhoeae*, *Chlamydia trachomatis*, *Trichomonas vaginalis*, Herpes Simplex Virus (HSV), Human Papilloma Virus (HPV), and Human Immunodeficiency Virus (HIV) [12,13]. Even in the absence of clinical symptomatology, women harboring CST-IV in vaginal microbiota may be at high risk of STI or of early pregnancy loss and pre-term delivery [20].

STI impose major health and economic burdens globally, in particular in low- and middle-income countries (LMICs) [23]. It is estimated that about 18.9 million people, of whom 48% aged 15 to 24 years, acquired a new STI every year [24]. This is likely an underestimation of the real incidence of STI, as they are often asymptomatic.

Sexually transmitted viral infections, the subject of this review, also represent a serious health problem globally. A total of 34 million people were living with HIV at the end of 2011. Worldwide prevalence of genital HPV infection is estimated at 440 million persons, causing 510,000 cases of cervical cancer and approximately 288,000 deaths [25]. An estimated 417 million people aged 15–49 (11%) worldwide have Herpes Simplex Virus type 2 (HSV-2) infection, and HSV-2 incidence was 23.6 million new cases per year [26]. Prevalence of HSV-2 infection was estimated to be highest in Africa (31.5%), followed by the Americas (14.4%). It increases with age, though the highest numbers of people newly-infected were adolescents [27].

Risk factors commonly associated with acquisition of STI include biological and behavioral factors, number of sexual partners, lack of information about transmission modalities, difficulty to access to prevention services and, as reported above, selected compositions of the vaginal microbiota [28]. Moreover, the epidemiology of these infections is extremely complicated by the fact that each of them increases the risk of contracting other STI.

Metagenomic studies largely contributed to define the microbial communities most frequently associated with these infections. The assessment of the interplay among microbial communities, immune cells reactivity and epithelial homeostasis has contributed to define the environment in which infections are established and, in particular for HPV infection, also the one that facilitates persistence of the pathogen. This information will be helpful for the development of new diagnostic assays as well as new therapeutic strategies based on the replacement of harmful microbial communities. The following sections describe in greater detail current knowledge on the interplay between vaginal microbiota, immune system and epithelial cells in the most prevalent sexually transmitted viral infections: HIV, HPV, HSV-2.

## 3. The Vaginal Microbiota and HIV Infection

The importance of understanding how bacterial communities modulate female genital health has come strongly into focus, in particular with the evolving knowledge about the influence of cervicovaginal microbiota on HIV acquisition. As known, the highest incidence of HIV infections, with an extraordinarily high number of infected women in reproductive age, is in the African continent and, particularly in the sub-Saharan regions.

Socio-economic factors largely contribute to such high incidence. The prevalence of CST-IV in the vaginal microbiota of African women, however, also suggested that this CST may affect the susceptibility to HIV infection. The molecular mechanisms that have been suggested are:

(A) A decrease in D-lactate concentrations and the lowering of virions trapping. In fact, d-lactate, the main metabolic product of numerous *Lactobacillus* species, including *L. crispatus*, contributes to trap virions by favoring hydrogen bridges between HIV surface proteins and carboxylic groups of mucins [29].

(B) CST-IV microbes produce enzymes that degrade the mucus included sialidase, α-fucosidase, α-and β-galactosidase, *N*-acetyl-glucosaminidase, and glycine and arginine aminopeptidases [21,22,30].

(C) Antigen-presenting cells activated by bacterial products, in particular LPS, produce cytokines and chemokines which increase the recruitment of activated CD4^+^ lymphocytes [10].

The first observations on the associations between defined microbial communities and HIV susceptibility were obtained in cross-sectional studies. These studies have a serious limitation since the recruited people were at “high risk” of infection (e.g., sex workers) and left the doubt that changes in the microbiome may be a consequence of HIV infection rather than a contributing cause [10,31,32,33]. Recently, however, these doubts have been resolved: a prospective study on a cohort of healthy HIV-uninfected black South African women who were monitored for incident HIV infection clearly showed that none of the women who acquired HIV had a *L. crispatus* dominant community [34]; in contrast, women belonging to CST-IV experienced up to four-fold greater rates of HIV acquisition during follow-up compared to women of the CST-I, dominated by *L. crispatus* [34]. In particular, a subgroup of CST-IV dominated by *Prevotella bivia*, *Prevotella melaninogenica*, *Veillonella montpellierensis*, *Mycoplasma* species, and *Sneathia sanguinegens* was identified as the cervicotype with significantly higher risk of HIV acquisition [34]. Recent observations expanded the number of bacterial taxa associated with increased risk of HIV acquisition: in addition to *Prevotella* species, *Parvimonas* species type 1 and type 2, *Gemella asaccharolytica*, *Eggerthella* species type 1 and vaginal *Megasphaera* species were significantly associated with HIV acquisition [35].

The mechanisms underlying the increased HIV susceptibility likely reside in the ability of the bacterial taxa to induce a strong inflammation in the cervicovaginal environment with elevated concentrations of IL-17, IL-23 and IL-1β and high recruitment of CCR5^+^ CD4 T cells, the primary target of HIV infection. These cells also show an activated phenotype (HLA-DR+CD38^+^) and thus they are highly permissive for viral replication [34]. High numbers of activated γδ CD4 T lymphocytes expressing the Vδ2 chain, which potentially are targets of HIV and permissive for its replication, were also found increased in women with vaginal microbiota not dominated by *Lactobacillus* species [36], thus defining a framework where selected microbial communities are closely linked with immune activation and HIV infection.

Such knowledge also suggests that, in addition to vaccines, strategies to prevent HIV infection may include the stable colonization of vaginal microbiota with non-dangerous bacterial taxa to limit inflammation and T-cell recruitment. Since vaginal dysbiosis may undermine the efficacy of locally administered antiviral drugs [37], changes in vaginal microbiota will also improve the efficacy of these drugs.

## 4. The Vaginal Microbiota and HPV Infection

Infection with HPV is the most common viral infection of the reproductive tract and high-risk (HR) genital HPVs are central etiological agents in the development of cervical cancer and of its premalignant precursor, cervical intraepithelial neoplasia (CIN) [38]. HR-HPV genotypes 16, 18, 31, 33, and 35 are the most diffuse worldwide. Other HR genotypes are HPV 39, 45, 51, 52, 56, 58, 66 and 69 [39]. Generally, HPV infects the basal layer of the cervical squamous epithelium, where viral genomes persist as episomes at low-copy numbers [40]. High copies of virions are produced upon differentiation of epithelial cells and the new progeny of virions may be released from the epithelial surface. Infection of stem cell-like cells of the basal layer ensures persistence of infection [41].

The oncogenic activity of HR-HPVs is based on the functional properties of their E6 and E7 proteins [42]. These proteins inactivate p53 and retinoblastoma protein, respectively, and lead to the inhibition of apoptosis and to cell cycle progression of cells at the basal and differentiated layers of cervical epithelium. Viral integration with genetic alterations ultimately may induce uncontrolled cell proliferation [43,44]. At the histological level, different grades of squamous intraepithelial lesions are the result of persistent HR-HPV infection and, if undetected and untreated, they may lead to high-grade lesions and cancer in an average of 5 to 15 years [45].

The immune response to acute HPV infections is initially mediated by mucosal NK cells [46,47] and by epithelial cells which produce antimicrobial peptides with reported anti-viral effects [48]. However, HR-HPVs have evolved molecular strategies to escape innate and adaptive immunity [49,50,51,52]. HR-HPV infection never associates with a marked pro-inflammatory environment [53,54,55]. A rather high number of CD4^+^ CD25^+^ regulatory T cells and the presence of activated TH2 cells were reported in HR-HPV persistent infections and associated with the suppression of cytotoxic functions, induction of T cell anergy [56,57]. Consequently, virus-induced immune suppression may be responsible for increased infection with other sexually transmitted diseases such as *Chlamydia trachomatis* infection [58,59].

Emerging data support the notion that the vaginal microbiome is involved in the natural history of the disease [46].

Since HPV infections, even with HR genotypes, may be transient and more than 50% of them are cleared within six months while almost 90% are cleared within two years, we believe it is critical to consider data obtained in longitudinal studies and to devote special attention to the context in which they were obtained [4,46]. The first longitudinal study observed a cohort of 32 North American sexually active and premenopausal women over the course of 16 weeks using self-sampling at twice-weekly intervals [14]. A total of 930 samples of cervicovaginal mucous were obtained and microbiota studies revealed that women with CSTs III (dominance of *L. iners*) or with a subtype of CST-IV with a high proportion of *Atopobium*, *Prevotella*, *Gardnerella* species were most likely to be HPV positive and had the slowest rate of infection clearance.

In a subsequent survey, Di Paola et al. [30] used cervicovaginal samples collected in a study for HR-HPV screening program in Italy. A total of 55/1029 samples were positive for HR-HPV at the baseline screening. The authors used the results of the second HR-HPV screening performed after one year to stratify the baseline sampling in Clearance group, with no evidence of HR-HPV DNA after one year; the Persistence group, HR-HPV^+^ with at least one of the HPV-DNA genotypes of the baseline sampling after one year. A group of cervicovaginal samples from women negative for HR-HPV (HR-HPV^−^) infections was included as a control. The results of this study showed that a subgroup of CST-IV dominated by strictly anaerobic species (*Gardnerella*, *Prevotella*, *Atopobium*, *Megasphoera*) was prevalent in the Persistence group, compared with either the Clearance or Control group. Odds ratio analysis confirmed that this CST may be a risk factor for the persistence of HR-HPV infection.

In agreement with previous reports [60,61], a significant enrichment in *Sneathia* and *Megasphaera* was found in the group of HR-HPV^+^ women, compared to HR-HPV^−^ controls, while the *Atopobium* genus was significantly enriched in Persistence group, compared to the other groups.

Although the association of bacterial vaginosis with higher rates of HPV infection and persistence has been known from a long time [62], metagenomic data revealed that selected microbiota compositions increase the risk of infection, even in the total absence of clinical symptomatology. While it is quite clear that some microbial communities favor HPV infection by modifying the barrier effect of cervical mucus and of stratified epithelium, the effects of microbiota-related immunological changes on the outcomes of HPV infection are not elucidated yet. This may depend on the impact of HPV infection on the host’s immune defenses [53,54], and on the mucosal metabolism which can also affect the composition of the vaginal microbiota. Therefore, the immunological signature of HPV infection on vaginal microbiota has to be further elucidated and more data are needed to have a full understanding of how HPV infections are modulated by the vaginal environment.

Persistent infection with HR-HPV is a necessary, but not sufficient, condition for the development of cervical cancer. As soon as the establishment of HR-HPV infection occurs, cellular changes can be observed in the cervical exfoliated cells. Persistent infections can result in different grades of squamous intraepithelial lesions that ultimately lead to high-grade lesions and cancer in an average of 5 to 14 years if undetected and untreated [45].

Recently, much attention has been given to the role of vaginal microbiota in the progression of epithelial lesions up to carcinogenesis. Drago et al. [63] found a strong correlation between *Ureaplasma parvum*-HPV co-infection and CIN1. Mitra et al. [64] have studied a cohort of 169 women with different degrees of cervical lesions and showed that increasing severity of cervical lesions was associated with higher vaginal microbiome diversity and decreased relative abundance of *Lactobacillus* species. CST-IV was associated with increasing disease severity, irrespective of HPV status. [61,65]. In particular, *Atopobium vaginae* and *Lactobacillus iners* were associated with an increased risk of neoplastic progression in HPV^+^ women [61], while *Sneathia sanguinegens* [54,61,64] and other *Fusobacterial* species [61], *Anaerococcus tetradius* and *Peptostreptococcus anaerobius* [64], were shown to be associated with an increase in disease severity. Novel bacterial taxa (*Shuttleworthia*, *Gemella* and *Olsenella*) and *Streptococcus agalactiae* were found enriched in HPV^+^ women with low- and high-grade cervical dysplasia (LGD, HGD), invasive cervical carcinoma (ICC) [54]. *Sneathia* spp. was the only taxon significantly enriched both in HPV^+^ women without lesions of any grade and in women with precancerous lesions and cervical cancer, suggesting that this taxon may represent a metagenomic marker for CIN progression. In particular, *Sneathia* spp. were found enriched in precancerous groups, and invasive cervical cancer, particularly in subjects of Hispanic ethnicity [54].

The molecular mechanisms through which bacteria of CST-IV may favor neoplastic progression are: (a) they produce high levels of nitrosamines which are known carcinogens; (b) they induce DNA oxidative damage [66].

While HPV infection or clearance is not marked by a pro-inflammatory environment, patients with cervical dysplasia and ICC were shown to have an increase in pro-inflammatory cytokines and chemokines (TNF-α, TNF-β, MIP-1α, GM-CSF), as well as an increase in cytokines related to adaptive immune responses (IL-2, IL-4, SCD40L). [67]. Moreover, the concentrations of the immunomodulating cytokine IL-10 were found to be increased in the ICC group. However, no direct association between the increased concentrations of cytokines and chemokines and the microbiota composition was revealed. Only the concentration of IL36γ was always elevated in patients with carcinoma, suggesting a direct or indirect role of this cytokine in the carcinogenetic process [54]. These data strongly suggest that, similar to other mucosal sites, chronic genital inflammation may promote carcinogenesis.

In the future it is highly likely that metabolomic studies, using nuclear magnetic resonance and mass spectroscopy, may better define the association of vaginal microbiota composition in health and disease states. A better definition of the effects of individual bacterial species, such as *L. iners*, *A. vaginae*, and *Sneathia* species, on epithelial and immune cell functions is desirable. In fact, if the responsibility of a single bacterial species in HPV disease is defined, it will be possible to identify patients at higher risk using microchip array or metabolomic technologies. Moreover, it will be possible to develop new therapeutic strategies based on pre/probiotics, in order to manipulate the vaginal microbiota composition.

## 5. Herpes Simplex Virus Type-2 (HSV-2)

Herpes simplex virus type-2 (HSV-2) is a common STI worldwide and the leading cause of genital ulcer disease [27]. Most HSV-2 infections are asymptomatic, with >80% of HSV-2 seropositive individuals asymptomatically shedding virus. More women are infected with HSV-2 than men; in 2012 it was estimated that 267 million women and 150 million men were living with the infection. This is because sexual transmission of HSV is more efficient from men to women than from women to men [26].

After infection by sexual transmission, virus replication initiates in genital keratinocytes and may spread to thousands of cells. Then, the virus invariably enters the neuron to reach the dorsal root ganglia where lifelong latency is established [68]. Virus periodically reactivates within the ganglia and travels back down the neuron leading to either asymptomatic, low titer shedding, or less commonly recurrent ulcers which are typically associated with prolonged high titer shedding [69,70,71].

The first two studies examining the associations between HSV-2 and vaginal microbiota were published in 2003 [72,73]. The main data emerging from these studies was that lack of *Lactobacillus* protective species and a history of BV was significantly associated with HSV-2 seropositivity [72]. Additionally, prospective studies confirmed the association between prevalent HSV-2 infection and BV [74,75]. In the case of HSV-2 infection, the association with vaginal dysbiosis is bi-directional. HSV-2 infection indeed seems to increase the occurrence of dysbiosis by multiple mechanisms: (a) the intermittent HSV-2 reactivation also leads to immune activation in the genital environment favouring changes in microbiota composition [76]; (b) increased iron availability which facilitates the growth of *Gardnerella vaginalis* [77]. In the same way, BV increases genital shedding of HSV-2 [73,78,79].

HSV2 infection was extensively studied because it represents a high risk factor for HIV acquisition [80]. This increased risk may occur because HSV-2 reactivation disrupts the epithelial barrier and recruits activated CD4 cells, which are target cells for HIV infection, into the lesion. Moreover, it has been shown that the HSV regulatory proteins ICP0, ICP4, VP16 upregulate HIV replication and increase the frequency and the titer of mucosal HIV shedding. This may occur during both clinical and asymptomatic HSV reactivation. [81,82].

Microbiota-HSV2 interactions and the corresponding changes in vaginal immunity were recently studied through NGS technologies in a group of 51 African Caribbean and other black (ACB) HIV^−^ women [83]. The authors found that HSV-2 infection was associated with an increased number of mucosal CD4^+^ T-cell subsets without significant alterations in local proinflammatory cytokines; selected cytokines or antimicrobial peptides (MIP-3α, IL-6, IL-8, LL-37, and HNP1-3), associated with the number of CD4+ T cells and these were distinct from cytokines usually associated with CST-IV, suggesting that HSV-2 and vaginal CST-IV enhance HIV susceptibility through independent mucosal immune mechanisms.

In murine experimental models of HSV-2 infection, Oh et al. investigated the mechanisms by which commensal bacteria elicit immune protection against HSV-2 infection of the vaginal mucosa [84]. The authors demonstrated that vaginal dysbiosis, like that induced by antibiotic therapy, affects the T-cell mediated adaptive immune response inducing a marked suppression. The molecular mechanisms at the basis of immune-suppression relied on high concentrations of IL-33, an alarmin secreted by damaged epithelial cells in the vaginal environment [85] after antibiotic treatment.

IL33 strongly inhibits the IFN-γ secretion by activated mucosal T cells, limiting the recruitment of T cells which is required to clear viral infection. The suppressive effects of IL33 are also mediated by a recruitment of innate lymphocytes type 2 and eosinophils into vaginal tissue, which contributes to suppression of antiviral immunity against mucosal HSV-2 infection by producing cytokines interfering with a TH-1 response [86]. Interestingly, protease activity by commensal bacteria resistant to antibiotic-treatment is likely responsible for epithelial damage and IL-33 secretion. Thus, in the case of HSV-2 infection a complex interplay among the virus, immune cells, and microbiota occurs, and it likely increases the susceptibility to other sexually transmitted diseases, including HIV.

## 6. Gaps and Challenges

Despite recent advances, significant gaps in knowledge concerning the vaginal microbiota have yet to be solved. Prevention strategies of sexually transmitted viral infection should be mainly based on the prevention and or treatment of recurrent BV. Treatment with oral or vaginal metronidazole or clindamycin is typically efficacious in the short term (as defined by Nugent or Amsel criteria) but recurrence rates are high [87]. An efficient strategy in the longer term may specifically target pathobionts or BV-associated anaerobs, while sparing *Lactobacillus* species or colonizing vaginal microbiota with appropriate probiotics. Common vaginal strains as *L. gasseri* and *L. crispatus* have been used in recurrent BV with moderate results; indeed, few randomized trials only (all including *Lactobacillus rhamnosus*) showed lower rates of recurrent BV in the probiotic group [88]. Hormonal therapy offers a potential approach to promote *Lactobacillus* dominant communities [89] while treatments to maintain vaginal acidity were not able to promote a healthy vaginal microbiota [90]. Treatments specifically targeting dysbiosis-associated anaerobes or pathobionts while sparing *Lactobacillus* species combined with biofilm disrupting agents, systemic or topical estrogen, and/or *Lactobacillus*-containing vaginal pro- or synbiotics might be more efficacious in the longer term.

The definition of cervicovaginal bacteria which mediate immunomodulatory mechanisms will help to identify molecules and to set up therapeutic strategies for infections that take advantage from a vaginal inflammatory environment. Finally, even when effective diagnostic tools are developed, it will be necessary to define the “harmful” levels of cervicovaginal inflammation, the appropriate "timing" of the therapeutic intervention and which women to target. We are confident that the progress achieved in recent years will make it possible to find the optimum solutions for these issues.

## Abbreviations

CVF	Cervicovaginal fluid
CST	Community State Type
STI	Sexually transmitted infections
HIV	Human immunodeficiency virus
HR-HPV	High risk human papilloma virus
HSV2	Herpes simplex virus 2
